# The stability of envelope-pseudotyped lentiviral vectors

**DOI:** 10.1038/s41434-020-00193-y

**Published:** 2020-09-24

**Authors:** Iris J. C. Dautzenberg, Martijn J. W. E. Rabelink, Rob C. Hoeben

**Affiliations:** Section of Virus and Stem Cell Biology, Department of Cell and Chemical Biology, Leiden University Medical Center, Albinusdreef 2 S1-P, PO Box 9600, 2300 RC Leiden, The Netherlands

**Keywords:** Genetic vectors, Gene therapy

## Abstract

Lentiviral vectors have become popular tools for stable genetic modification of mammalian cells. In some applications of lentiviral vector-transduced cells, infectious-lentiviral particles should be absent. Quantification of the free-vector particles that remain from the inoculum can be difficult. Therefore a formula was established that yields an estimation of the ‘Reduction Ratio.’ This ratio represents the loss of titer based on a number of vector-inactivating effects. In this study, we evaluated several parameters and assumptions that were used in the current formula. We generated new data on the stability and trypsin sensitivity of lentiviral vectors pseudotyped with eight heterologous envelope proteins and the loss of vectors by washing or passaging the cell cultures. Our data demonstrate that the loss of virus titer under the influence of trypsin as well as the half-life of the particles in tissue culture medium is dependent on the vector’s envelope protein. While VSV-G-envelope-pseudotyped particles were unsensitive to trypsin, the titer of vectors pseudotyped with other envelope proteins decreased 2–110-fold. The half-life in culture medium ranged from 8 to 40 h for the different envelope-pseudotyped vectors, with 35 h for VSV-G-envelope-pseudotyped vector particles. Additionally, we found that removal of the culture medium from Ø35 mm to Ø10 cm dishes reduces the amount of vector particles in the culture by 50-fold and 20-fold, respectively. Together these data can be used to more precisely estimate the maximum number of free lentiviral vector particles in cell cultures.

## Introduction

Lentiviral vectors have become the method of choice for many applications that require stable genetic modification of mammalian cells in cell culture [1]. Lentiviral vectors have been predominantly derived from human immunodeficiency virus 1 (HIV-1). The popularity of lentiviral vectors stems from their propensity to integrate their genetic payload into the chromosomal DNA of their host cell. They do so in cycling cells as well as in mitotically quiescent cells.

There is a variety of systems for the production of lentiviral vectors. With the more recent 2nd and 3rd generation production systems, batches of replication-defective lentiviral vectors can be produced that are free of contaminating replication-competent lentiviruses [2, 3]. So far, there are no reports that describe the generation of replication-competent lentivirus during the production of replication-defective lentiviral vector stocks with the 2nd and 3rd generation production systems [4].

While the HIV virus itself depends on the expression of the CD4 molecule on the surface of the target cell for productive infection, the lentiviral vectors are usually produced with an alternative envelope protein in their lipid membrane. The envelope of the lentiviral particle is ‘pseudotyped’ with the alternative envelope glycoprotein [5]. This allows the transduction of cells lacking CD4 when the pseudotyped envelope protein engages its cognate receptor on the target cell. A range of different envelope proteins have been employed for production of envelope-pseudotyped lentiviral vectors. These include the envelope glycoprotein of vesicular stomatitis virus (the VSV-G protein), the envelope protein of nonhuman retroviruses (e.g., the ecotropic retrovirus murine leukemia virus (MULV), the gibbon ape leukemia virus (GALV), the feline endogenous RD114 retrovirus, Moloney MULV 4070A, Moloney MULV strain 10A1, as well as the rabies virus glycoprotein, and the measles virus hemagglutinin and fusion glycoproteins [6–14]. All but one of these viruses is capable of binding receptors on human cells: solely the ecotropic MULV recognizes a receptor on murine cells only.

The VSV-G protein has been the most popular for pseudotyping the envelope proteins of lentiviral vectors. VSV-G binds LDL-Receptor family members, allowing these viruses to infect a wide range of cell types of many distinct host species, including rodent and human cells [15]. The others have been used predominantly for transduction of human blood and bone-marrow derived cells, as in these cells VSV-G-pseudotyped vectors are less effective [16]. The ecotropic MULV-envelope-pseudotyped vectors have been used mainly for experiments in which lentiviral-vector transduction is to be limited to murine cells [17].

The so-called self-inactivating or SIN lentiviral vectors are integrated in the host cell chromosome in a predictable structure in which the major part of the U3 regions in the long terminal repeats (LTRs) are lost. As a result, the LTRs cannot serve as a promotor and there is virtually no transcription initiating in the SIN LTR. As a consequence, the expression of the transgene is dependent on a heterologous promoter inserted between LTRs. Transcription initiating at the internal promotor does not generate mRNAs that harbor the lentiviral packaging signal. Consequently, in the absence of transcripts that harbor the packaging signal the integrated lentiviral vector cannot be mobilized upon superinfection by replication-competent lentiviruses [2].

A key safety factor of lentiviral vectors is the resistance of the 2nd and 3rd generation production systems to the formation of replication-competent lentiviruses during production. These production systems allow the production of lentiviral vector stocks that are free of contaminating replication-competent lentiviruses [2, 18, 19]. It goes without saying that the production and handling of lentiviral vector particles, and the handling of cells modified with such vectors, can be associated with (environmental) risks and therefore the use of these vectors is subject to Genetically Modified Organisms (GMO) guidelines and regulations. As lentiviruses are pathogens, handling lentiviral vectors and lentiviral vector-modified cells requires the use of biosafety facilities. In contained-use applications the handling of lentiviral vector stocks produced with 2nd or 3rd generation vector lots usually requires biosafety level-2 (BSL-2) containment. This is based, amongst others, on the resistance of the production systems to generation of replication-competent lentiviruses by genetic recombination. The absence of detectable mobilization of SIN vectors by replication-competent lentiviruses has led to a practice in which the culture and maintenance of lentiviral vector-transduced cells is permitted under BSL-1 conditions once the vector particles added as inoculum to the cells are removed or inactivated. Therefore in many countries the use of HIV-derived lentiviral vectors is permitted under BSL-2 conditions and the cells modified with such vectors can be handled under BSL-1 conditions once free lentivirus-vector particles are absent [20].

There are few risk factors associated with handling lentivirus-vector particles that should be considered. With the vector production systems used to date, in which the helper functions are separated on three or more different plasmids, the risk of forming replication-competent lentiviruses during production has been eliminated. However, even in the absence of replication there is a risk of insertional mutagenesis [21]. The use of SIN vectors considerably reduces the chance that this leads to transcriptional activation of neighboring genes and clonal expansion of transduced cells. In a retrospective study of recipients of SIN lentivirus-vector-modified stem cells, Fischer et al. reported the absence of selective clonal expansion of particular gene modified clones in all of 33 recipients of SIN-retroviral vector-modified cells [22].

However, this does not eliminate the potential negative consequences of overexpression of the transgene. Therefore it remains essential to take the transgene into account when evaluating the biological safety of the free lentivirus-vector particles. Vectors with genes encoding toxic or transforming products deserve special attention. To obtain permission for working under BSL-1 with lentivirus-transduced cells a stepwise (environmental) risk assessment (ERA) is required. A key step in the ERA is assessment of the risks that may be associated with free replication-defective viral particles that may remain from the initial vector inoculum used for generating the genetically modified cell product.

Several years ago the Commission on Genetic Modification (COGEM) in the Netherlands proposed a formula that could be used as an aid for estimating the maximal amount of residual free infectious viral particles in the cell product to be evaluated [20]. This formula should assist risk analyses for standard lentivirus-transduction in the absence of actual experimental data. COGEM’s formula calculates the reduction in the amount of residual free infectious viral particles as function of the time, the number of vector-inactivating steps, and the number of washing steps:

Reduction Ratio = (20^*W*^ **×** 200^*I*^ × 2^2.4*T*^)/*C*_*i*_.

In the formula, the parameter ‘*W*’ represents the number of times the cell culture was washed, ‘*I*’ signifies the number of inactivating washes with trypsin or human serum, ‘*T*’ is the culture time after the start of transduction in days. The factor 2.4 is based on the published half-life of VSV-G-envelope-pseudotyped infectious-lentiviral vector particles at 37 °C. ‘*C*_*i*_’ is the measured amount of infectious viral particles in the inoculum.

The Reduction Ratio represents the fold-decrease in the concentration of free infectious vector particles related to the amount of infectious viral vectors added to the culture. A ratio of 1 indicates that, based on the procedure used, the amount of free infectious virus particles present is reduced to 1. A Reduction Ratio of 100 indicates that 100 times more viral particles are expected to be inactivated than were initially present in the inoculum, meaning that a maximum of 0.01 infectious particles are left on average in the culture.

This reduction-ratio approach has more recently also been used in a European document ‘Good Practice on the assessment of GMO-related aspects in the context of clinical trials with human cells genetically modified by means of retro/lentiviral vectors,’ which was endorsed by the national competent authorities of Austria, Belgium, Cyprus, Czech Republic, Denmark, Estonia, Finland, France, Germany, Greece, Hungary, Ireland, Italy, Latvia, Luxembourg, Malta and the Netherlands [23].

In this study, we performed experiments to expand the range of conditions in which the formula can be applied. We determined the necessary parameters in a panel of envelope-pseudotyped lentiviral vectors and included VSV-G-pseudotyped vectors as comparator. Also we verified the literature data on which the current parameter values are based.

We performed the following analyses: we assessed the half-life in cell culture medium of lentiviral vector particles pseudotyped with other envelope proteins for which no half-life data are known in the literature, and we sought to confirm published half-life data for the VSV-G envelope glycoprotein. Also we determined the efficiency of trypsin-mediated vector inactivation for the envelope-pseudotyped vectors, and measured the amount of residual medium for two dish sizes, as a guide to estimate the efficiency of washing the cell cultures. In addition, we determined the effect of variations in the infection procedure on the apparent infectious viral particle titers and measured how polybrene, often added to the culture medium for enhancing the efficiency of vector transduction, affects the vector half-life.

The outcomes of our study provide new parameters that can be used to estimate the amount of residual infectious vector particles for envelope-pseudotyped lentiviral vectors. Our data should aid estimating the maximal amounts of residual lentivirus vectors that remain from the inoculum to which the target cells have been exposed.

## Materials and methods

### Acquiring the plasmids encoding the virus envelope proteins

Plasmids containing the gene for VSV-G glycoprotein (pCMV-VSV-G, #8454), rabies virus (strain SAD B19) glycoprotein (pHCMV-RabiesG, #15785), ecotropic Moloney MULV-envelope gene (pHCMV-EcoEnv, #15802), Moloney MULV strain 10A1 envelope gene (pHCMV-10A1, #15805), and amphotropic 4070A gene (pHCMV-AmphoEnv, #15799) were purchased from Addgene (www.addgene.org) with their respective plasmid numbers. Addgene is a nonprofit plasmid repository and plasmids are provided by researchers. Plasmid pCMV-VSV-G was a kind gift from dr. Bob Weinberg. All other Addgene derived plasmids were a kind gift from Dr. Miguel Sena-Esteves. The plasmids encoding the GALV-envelope protein (phCMV-GALV TR) and the feline endogenous virus RD114-envelope glycoprotein (phCMV-RD114 TR) were kind gifts from Professor Frank Staal (Leiden University Medical Center, Leiden, The Netherlands). Plasmids encoding the H and F glycoproteins of measles virus strain Edmonston (pCG-HD24 and pCG-Fdel30) were kindly donated by professor François-Loïc Cosset (Claude Bernard University, Lyon, France). The helper plasmids encoding HIV-1 gag/pol (pMDLg-RRE, #12251) and HIV-1 rev (pRSV-REV, #12253) were purchased from Addgene and were a kind gift of professor Didier Trono (School of Life Sciences, Ecole Polytechnique Fédérale de Lausanne (EPFL), Lausanne, Switzerland). Plasmid pRRL-cPPT-CMV-GFP-PRE-SIN (here named pLV-CMV-GFP) was a kind gift of Dr. Jurgen Seppen (Amsterdam UMC, location AMC, Amsterdam, The Netherlands).

### Production of lentiviral vectors

Third generation self-inactivating lentiviral transfer vectors containing a green fluorescent protein (GFP) transgene, the plasmids encoding the different envelope proteins and the two other helper plasmids (encoding HIV-1 gag/pol, HIV-1 rev) were co-transfected overnight into 60–70% confluent 293T cells (human Ad5-E1 transformed embryonic kidney cells containing expressing the Simian Virus 40 (SV40) T-antigen gene, obtained from ATCC, Manassas, Virginia, USA) using the polyethyleneimine (PEI) method as described before [24]. All cell types used in this study were regularly tested for mycoplasma contamination, according to our standard laboratory protocol. Briefly, plasmids were mixed in the following concentration: 7.5 µg [envelope gene], 11.4 µg pMDLg-RRE, 5.4 µg pRSV-REV and 13.7 µg pLV-CMV-GFP followed by the addition of Opti-MEM^®^ I (Gibco; Thermofisher Scientific, Breda, The Netherlands catalog #31985070) to a total volume of 1 ml. In a second tube, Opti-MEM^®^ was added to 114 µl PEI (1 mg/ml) to a total volume 1 ml and mixed. For obtaining lentiviral vectors with the envelop proteins of measles virus, 6.23 µg of each of the plasmids pCMV-HD24 and pCMV-Fdel30 were added to the mixture, for the other plasmids the quantities were as listed above. The content of the two tubes was gently mixed together, incubated at room temperature (RT) for 10 min and added to the culture medium (Dulbecco’s Modified Eagle Medium, Gibco|Thermofisher Scientific, catalog #41966052, supplemented with 8% FCS (Biowest, Nuaillé, France, catalog #S1810-500) of a T175 flask containing 293T cells. After overnight incubation (37 °C/5% CO_2_) the culture medium was replaced and supernatant was harvested after 48 and 72 h post transfection, centrifuged for 5 min at 845 × *g* at RT and subsequently passed through 0.45 µm pore-sized filters (Supor PES, Acrodisc, Supor polyethersulfone membrane, pore size 0.45 µm, diameter of filter unit 25 mm, catalog #4614, Pall, Medemblik, The Netherlands), aliquoted and stored at −80 °C. The lentivirus-vector concentrations were quantified by antigen capture ELISA measuring HIV p24 levels (HIV-1 p24 Antigen ELISA 2.0, catalog #0801008, ZeptoMetrix Corporation, NY, USA).

Concentrated lentiviral vector stocks were obtained by ultracentrifugation of the filtered vector-containing culture medium. In total, 31 ml of culture medium was added to a 38.5 ml polyallomer tube (Beckmann Coulter, Woerden, The Netherlands, 25 × 89 mm, catalog #326823) onto a 4 ml 20% sucrose solution, placed into a Beckmann Sw32 Ti rotor and centrifuged at 50,000 × *g* for 2 h at 4 °C. Subsequently, the supernatant was removed and the pellet was resuspended in 0.6 ml T_50_N_130_E_1_ buffer (50 mM Tris-Cl, 130 mM NaCl and 1 mM EDTA; pH 7.8) by gently shaking overnight at 4 °C, aliquoted, and stored at −80 °C.

### Residual culture-medium quantitation on culture dishes

Dry and empty Ø35 mm or Ø10 cm (Ø 35 mm dishes: Greiner CELLSTAR^®^, Alphen aan den Rijn, The Netherlands, catalog #P6987/627160, Corning-Falcon, Glendale, Arizona, USA catalog # 353001, and Stem Cell Technologies, Köln, Germany, catalog #27100. In total, Ø10 cm dishes: Greiner CELLSTAR^®^, catalog #7612/664160) culture dishes were weighed on an analytical balance before adding resp. 3 and 10 ml culture medium supplemented with 8% FCS, penicillin-streptomycin (Gibco|Thermofisher Scientific, catalog #15140122) and 8 μg/ml polybrene (Sigma Aldrich, catalog #10768-9). Culture medium was aspirated by a resp. 5 and 10 ml serological pipette (Greiner CELLSTAR^®^, Sigma Aldrich, catalog # resp. 7615 and 7740) at an angle of 45°, avoiding to touch the bottom of the dish, until the point that the dish was visually empty and dishes were weighed again. Next, the volume of culture medium was added again to the dishes and removed by a custom-made platinum aspiration needle coupled to a vacuum system, in the same manner as described before, and dishes were weighed again. The density (specific weight) of the applied culture medium was determined by weighing 1 ml of culture medium in a microcentrifuge tube on an analytical balance. From the residual weight of the culture dishes and the density of the culture medium, the residual volume in the culture dishes was calculated. We performed a single experiment on every described dish size and brand and examined ten dishes per experiment.

### Lentiviral-vector transduction of 293T and B77 cells

GFP-transgene containing lentiviral vectors pseudotyped with the ecotropic Moloney MULV-envelope protein were assayed on B77 cells (kindly donated professor Dinko Valerio, Leiden University Medical Center, department of Cell and Chemical Biology, Leiden, The Netherlands), which are nonproducer B77 avian sarcoma virus transformed BALB/3T3 mouse embryonic cells [25]. All other envelope-pseudotyped lentiviral vectors were assayed on 293T cells [26] in 24-well format. Cell culture medium was supplemented with 8% FCS and penicillin-streptomycin in all performed experiments. By flow-cytometry analyses, the percentage of cells displaying GFP signal was assayed compared to non-transduced control cells. In these cell-based assays (the assays for determining the half-life, trypsin sensitivity, and the degree of cellular uptake of the different envelope-pseudotyped vectors) the quantity of vector particles that were being added were based on volume. In these assays, we aimed at a maximum of 30–40% GFP-positive cells to ensure that the majority of cells is transduced by a single lentiviral vector particle. The p24 titer of the vector stocks was determined for comparison purposes only as it was impossible to correlate the physical particle titer to the biological activity of a vector stock with this method.

### Trypsin inactivation assay

Small volumes of concentrated envelope-pseudotyped lentiviral vectors (5–15 µl) were incubated with 60 µl 0.05% trypsin-EDTA (from 0.5% Trypsin-EDTA, Gibco, ThermoFisher Scientific, catalog # 15400054) in PBS or PBS only in microcentrifuge tubes. After incubation for 5 min at 37 °C trypsin was inactivated by the addition of 600 µl culture medium containing 8% FCS, supplemented with 8 µg/ml polybrene and divided over 2 wells containing 60–70% confluent 293T or B77 cells (24-well format, 300 µl/well). Culture plates were centrifuged for 90 min at 845 × *g* at 33 °C, 300 µl fresh culture medium was added and cells were incubated at 37 °C/5% CO_2_ for 2 days before flow-cytometry analysis. We performed two independent experiments with technical duplicates for VSV-G-, rabies-, GALV- and 4070A-envelope-pseudotyped vectors. For measles-, RD114- or MULV-envelope-pseudotyped vectors, three independent experiments with technical duplicates were done. Error bars in the graph (Fig. 2) represent ± standard deviation of the independent experiments.

### Half-life assay

The indicated volume of unconcentrated or concentrated envelope-pseudotyped lentiviral vectors was added to 250 µl culture medium into a 24-well culture plate and incubated at 37 °C/5% CO_2_ for the indicated time. Subsequently, to sample that were incubated in the absence of polybrene, 10 µl culture medium containing 208 µg/ml polybrene was added (to obtain a final concentration of 8 µg/ml polybrene). The vector-containing medium was transferred to 60–70% confluent 293T or B77 cells and centrifuged for 90 min at 845 × *g* at 33 °C. Next, 250 µl fresh culture medium was added and cells were placed at 37 °C/5% CO_2_. Two days after transduction cells were harvested for flow-cytometry analyses. From the graphical representation of the percentage of GFP-positive cells obtained by flow cytometry, the half-life was determined following the formula for exponential decay *N*(*t*) = *N*0 × *e*^−*λt*^, in which *t*_½_ = ln2/*λ*. The experiment that examined the influence of polybrene on the half-life of the vectors is performed once. Error bars in the graph (Fig. 3a) represent ± standard deviation of technical triplicates per time point. The half-life of the envelope-pseudotyped lentiviral vectors is determined in three independent experiments for MULV- and RD114-envelope-pseudotyped vectors and two independent experiments for all other vectors. The error bars in the graph (Fig. 3b) represent ± standard deviation of technical triplicates per time point.

### Residual lentiviral vectors in culture medium/cellular uptake assay

The indicated volume of unconcentrated or concentrated envelope-pseudotyped lentiviral vector was added to 250 µl culture medium supplemented with 8 µg/ml polybrene into a 24-well culture plate with or without 60–70% confluent 293T or B77 cells. Subsequently, cells were centrifuged in the 24-well plate for ‘spinfection’ [27] or only incubated at 37 °C/5% CO_2_. Vector-containing medium in culture plates without cells were placed at 37 °C/5% CO_2_ without centrifugation. After 90 min 250 µl fresh culture medium was added to all wells and plates were incubated at 37 °C/5% CO_2_. The next day, the culture medium was replaced and vector-containing medium was transferred to fresh cells again followed by a spin infection as described above. Two (secondary infection) or three (primary infection) days after transduction cells were harvested for flow-cytometry analyses. Tables 4–6 each present single experiments with technical triplicates.

### Flow-cytometry analysis of lentiviral vector-transduced cells

Cells were harvested in PBS and subsequently fixed in 4% paraformaldehyde for flow-cytometry analyses, resuspended in flow-cytometry buffer (0.5% BSA and 2 mM EDTA in PBS), and assayed on a BD LSRII flow cytometer. Per sample at least 10,000 living cells/events were measured and data were analyzed using FlowJo™ software version 10 (Becton Dickinson, Vianen, The Netherlands).

### Statistical analyses

General standard deviations of duplo of triplo values were calculated using the stdev function in Microsoft Excel. Standard deviations of the reduction factors, consisting of a ratio between two values with their own standard deviations, were calculated using the formula:

$${\it{\updelta }}{R} = \left| {R} \right| \times \sqrt {\left( {\left( {{\it{\updelta }}{X/X}} \right)^{2} \, + \, \left( {{\it{\updelta }}{Y/Y}} \right)^{2}} \right)}.$$

In this formula, *δR* represents the standard deviation of the average reduction factor; |*R*| means the average absolute value of the reduction factor; *δX* expresses the standard deviation of the numerator of the reduction factor ratio; *X* signifies the average value of the numerator of the reduction factor ratio; *δY* symbolizes the standard deviation of the denominator of the reduction factor ratio; and *Y* is the average value of the denominator of the reduction factor ratio.

The standard deviations of the average reduction factors or percentage of residual vector activity of different experiments were calculated with the following formula:

$$\delta {R} = \!\left( \!\left( {\left( {\left( {{n}_{a}{ \, - \, 1}} \right) \times \delta {X}^{2}} \right) + \left( {{n}_{b} - {1}} \right) \times \delta {Y}^{2}} \right)/ \left( {{n}_{a} - {1}} \right) +\left( {{n}_{b} - {1}} \right) \right).$$

Here, *δR* represents the standard deviation of the average reduction factor; *n*_*a*_ is the number of samples in the first experiment with average value *X*; *δX* indicates the standard deviation of average value *X*; *n*_*b*_ signifies the number of samples in the second experiment with average value *Y*; and *δY* represents the standard deviation of average value *Y*.

## Results

### Residual culture medium volume on culture dishes

**Fig. 1:**
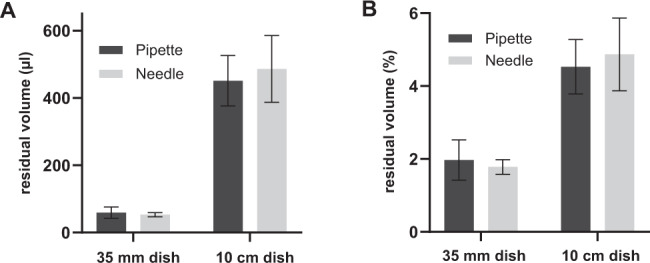
Residual volume of culture medium in culture dishes of frequently used dimensions. Residual volume of complete culture medium in µl (**a**) or as percentage of the input volume (**b**) on Ø35 mm and Ø10 cm dishes after aspiring the medium with a pipette or aspiration needle. Error bars represent ± standard deviation (*n* = 10 dishes).

When the cell culture medium of adherent cells is replaced, a limited amount of medium is retained in the dish. We measured the residual culture medium volume retained on culture dishes of 35 mm and 10 cm in diameter, as these are most commonly used in the laboratory. To pre-weighed dishes, 3 ml and 10 ml of culture medium was added on Ø35 mm and Ø10 cm culture dishes, respectively (*n* = 10). After aspiring the medium with either a disposable pipette or a custom-made platinum aspiration needle coupled to a vacuum system the dishes were weighed again and the residual weight of the medium was converted to the residual volume (Fig. 1a) and the percentage of the input weight (Fig. 1b).

The residual volume on Ø35 mm dishes was on average 59 ± 17 μl and 53 ± 6 μl when the culture medium was aspirated with a pipette or an aspiration needle respectively and 451 ± 75 μl and 486 ± 99 μl on Ø10 cm dishes. Considering the standard deviation, this indicated that the residual volume was equal with both aspiration methods. The presented results were obtained on dishes from Greiner CELLSTAR^®^. We performed the same experiment on Ø35 mm dishes from other suppliers (Corning-Falcon and Stem Cell Technologies) and similar to the results with the Greiner CELLSTAR^®^ dishes, we observed no differences in the residual volumes on this dishes between both aspiration methods (data not shown). Similarly, we noted that the inclusion of polybrene in the medium did not affect the amount of residual medium in the dishes after aspiration (data not shown). These data demonstrate that neither the brand of cell culture dishes, not the presence of polybrene affect the residual amount of medium.

On Ø35 mm dishes on average respectively 2.0 ± 0.6% and 1.8 ± 0.2% of the input volume was left on the dish after aspiration of the medium with a pipette or an aspiration needle. This would imply a 50-fold reduction of the amount of free lentiviral particles upon replacement of the culture medium. The residual volume on Ø10 cm dishes was on average respectively 4.5 ± 0.8% and 4.9 ± 1.0% of the input volume after aspiration with pipette or needle. This calculates to a 20-fold reduction in the amount of free virus upon medium replacement for cell cultures in Ø10 cm dishes. These data demonstrate that the reduction in the amount of free virus upon medium replacement, or upon washing the cells, is dependent on the size of the culture dishes.

### Trypsin inactivation of envelope-pseudotyped lentiviral vectors

To determine the decrease in concentration of free infectious-lentiviral vector particles in the cell culture medium upon trypsin treatment, we generated batches of lentiviral vectors pseudotyped with each of eight different envelopes (Table 1). In each batch, the same lentiviral vector was used that is equipped with an enhanced GFP-transgene driven by an internal CMV immediate early enhancer/promoter to monitor its capacity to transfer genes into susceptible cells.

**Table 1:** Envelope-pseudotyped lentiviral vectors studied in this project.

Full name of envelope protein	Abbreviation
Vesicular stomatitis virus G protein	VSV-G
Maesles virus hemagglutinin and fusion glycoproteins	Measles
Gibbon ape leukemia virus envelope protein	GALV
Rabies virus glycoprotein	Rabies
Feline endogenous virus RD114-envelope glycoprotein	RD114
Moloney murine leukenia virus 4070A-envelope protein (amphotropic)	4070A
Moloney murine leukemia virus strain 10A1 envelope protein (amphotropic)	10A1
Moloney murine leukemia virus envelope glycoprotein (ecotropic)	MULV

The enzyme trypsin is routinely used in the laboratory to dissociate attached cells from their culture dishes to be able to apply a treatment to the cells. Trypsin is an endopeptidase cleaving C-terminal of lysine and arginine residues in polypeptides. The enzyme is capable of cleaving the envelope proteins on lentiviral vector particles, thereby inactivating free floating particles in solution and dissociating bound, extracellular particles from cells, leaving them unable to transduce the target cells and ready to be washed away. In the our study we investigated the effect of trypsin on the eight envelope-pseudotyped lentiviral vector particles. We incubated a small volume (5–15 µl) of purified serum-free particles with 60 µl 0.05% trypsin-EDTA in PBS (or, as control, PBS only) for 5 min at 37 °C to mimic the conditions that are used in the cell culture. Trypsin was subsequently inactivated by adding DMEM with 8% FCS and the vector suspensions were added to 293T (or B77 for MULV) cell cultures to determine impact of trypsin treatment on the infectivity and GFP expression in the indicator cells.

**Fig. 2:**
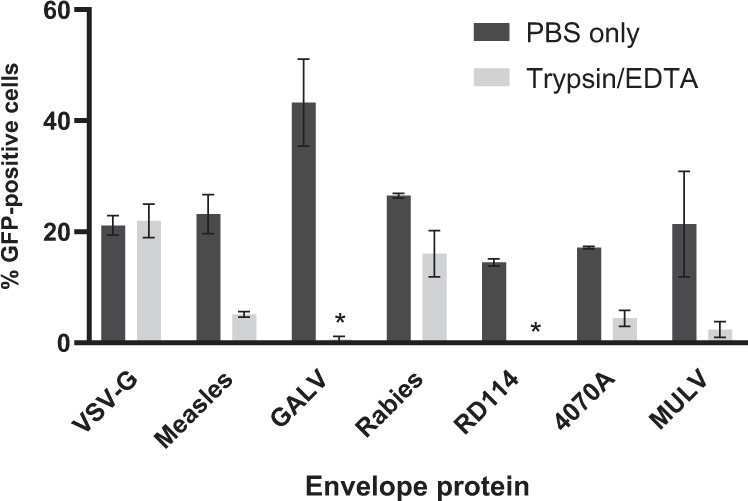
Inactivation of envelope-pseudotyped lentiviral vectors by trypsin treatment. The bars represent the mean percentage of GFP-positive cells upon transduction by trypsin-EDTA-treated or mock-treated (PBS only) lentiviral vectors (for 5 min, at 37 °C). Subsequently, pseudotyped lentiviral vectors were added to cells in FCS-containing cell culture medium. Two days post transduction cells were harvested and analyzed by flow cytometry. Error bars represent ± standard deviation of *n* = 2 independent experiments with technical duplicates. For measles-, RD114- or MULV-envelope-pseudotyped vectors, *n* = 3 independent experiments with technical duplicates. The 10A1-pseudotyped vector lost all GFP activity upon purification and was not included in these analyses. *The mean percentage of GFP-positive cells that were transduced with the trypsin-treated RD114- or GALV-pseudotyped lentiviral vector (resp. 0.13 ± 0.1% and 0.59 ± 0.6%) was below the representation limit of the graph.

The ability of trypsin and mock-treated envelope-pseudotyped lentiviral vectors to transduce cells is presented as percentage of GFP-positive cells (Fig. 2). While trypsin-treated VSV-G-pseudotyped vectors showed no decrease in activity compared with mock-treated vector particles, the activity of GALV- and RD114-pseudotyped lentiviral vectors was almost completely abolished upon trypsin treatment.

In addition, we represented the average residual activity of the vector particles by dividing the percentage of GFP-positive cells in the trypsin-containing condition by the GFP-positive cells in the PBS only condition (which was set at 100% activity). We also converted these data to a reduction factor as practiced in the formula (GFP + cells in PBS only/GFP + cells in PBS + trypsin) (Table 2). Please note that for splitting the cell cultures the cultures are washed once before trypsin treatment. This could be taken along in the formula.

**Table 2:** Residual activity and corresponding reduction factors of envelope-pseudotyped lentiviral vectors based on trypsin treatment.

Envelope protein	Residual activity (±SD)^a^ (%)		Reduction factor^b^
VSV-G	104	±15	1
Measles	23	±3	4
GALV	1	±1	79
Rabies	61	±11	2
RD114	1	±0.2	110
4070A	26	±15	4
MULV	10	±1	10

^a^The average percentage of residual envelope-pseudotyped lentiviral vector activity after trypsin treatment is calculated by dividing the % of GFP-positive cells transduced by trypsin-treated envelope-pseudotyped lentiviral vectors by the % of GFP-positive cells transduced by mock-treated vectors, ±standard deviation. *n* = 2 independent experiments with technical duplicates, for Measles, RD114 or MULV pseudotyped vectors, *n* = 3 independent experiments with technical duplicates (The 10A1-pseudotyped vector lost all GFP activity upon purification and was not included in these analyses.).

^b^The reduction factor based on the inactivation of the pseudotyped lentiviral vectors by trypsin is calculated by 100 divided by the percentage of residual activity after trypsin treatment.

The average reduction factor by trypsin of the different pseudotyped lentiviral vectors ranged from 1 (100% residual activity/no inactivation) up to 110 (≈1% residual activity). These data suggest that the trypsin sensitivity depends largely on the vector’s envelope protein.

### Half-life of pseudotyped lentiviral vectors

The infectivity of lentivirus-vector particles in tissue culture medium at 37 °C decreases with time. It is assumed here that the particles lose their infectivity based on a stochastic process with a constant half-life. Here we determined the half-life of the eight envelope-pseudotyped lentiviral vector particles under conditions that are representative for the daily routine in cell transduction experiments.

**Fig. 3:**
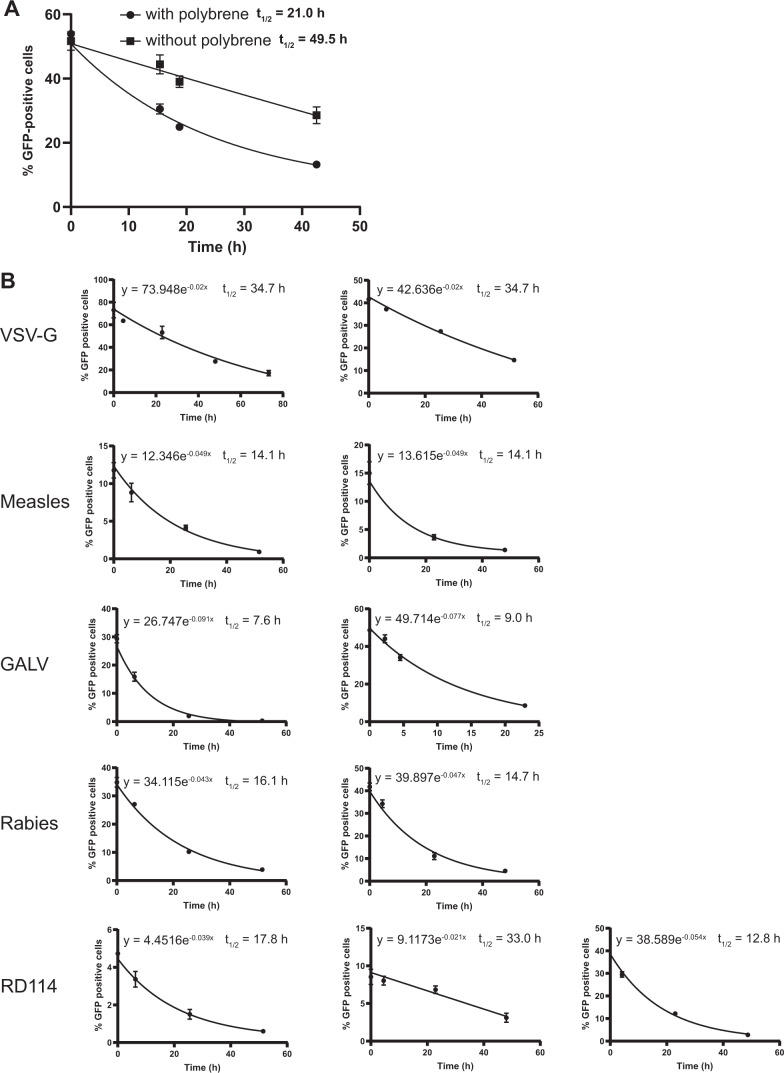

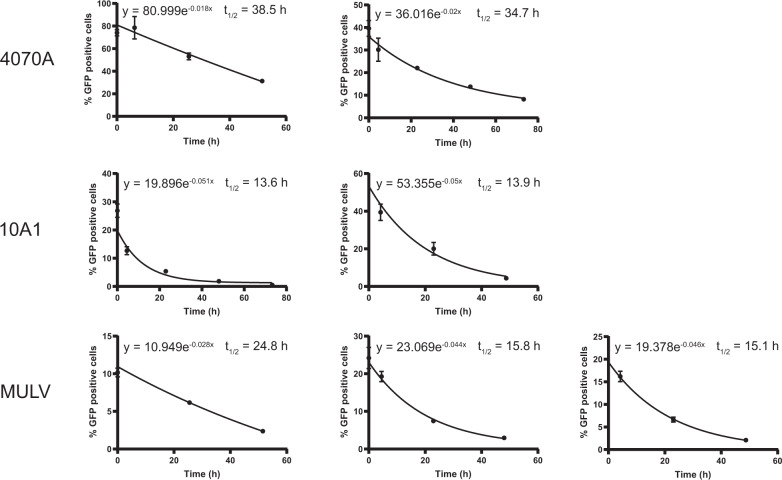
Half-life of envelope-pseudotyped lentiviral vectors. **a** Half-life of VSV-G-pseudotyped lentiviral vectors in the absence or presence of polybrene. Lentiviral vectors were incubated in culture medium at 37 °C with or without polybrene for the indicated time. At *t* = 0 all samples were added to cells simultaneously. Two days post transduction cells were harvested and assessed by flow cytometry. Error bars represent ±standard deviation of technical triplicates. **b** Half-life of eight envelope-pseudotyped lentiviral vectors. Lentiviral vectors were incubated in culture medium at 37 °C for the indicated time and processed further as above. Each graph represents an independent experiment, error bars in the graph represent ± standard deviation of technical triplicates. Some error bars in (**a**) and (**b**) could not be displayed as they were narrower than the data point.

Polybrene is routinely used to enhance the transduction efficiency of the lentiviral vector particles. In a pilot experiment we investigated the effect of polybrene on the half-life of VSV-G-pseudotyped particles by incubating the vector particles in FCS-containing culture medium (no cells) with or without polybrene for prolonged periods of time after which the vector-containing medium was transferred to cells. The data show that polybrene negatively affects the half-life of VSV-G-pseudotyped vector particles (Fig. 3a). Usually polybrene is absent during the culturing after transducing the cells. For these reasons we decided to study the half-life of the envelope-pseudotyped lentivirus-vector particles in absence of polybrene in the medium.

To assess the vector particle half-life lentiviral vector particles containing culture media were incubated at 37 °C (without cells) in a time series. At the end of the incubation time the vector-containing medium was added to 293T of B77 cells to determine the percentage of particles that is able to transduce the cells, leading to a measurable GFP expression. From the graphical representation of these data the half-life (*t*_½_) was determined following the formula for exponential decay *N*(*t*) = *N*0 × *e*^−*λt*^ in which *t*_½_ = ln2/*λ* (Fig. 3b). The reduction factors derived from the average half-life of the different vectors are presented in Table 3.

**Table 3:** Half-life and corresponding reduction factors of envelope-pseudotyped lentiviral vectors.

Envelope protein	Average HL		Reduction factor
	(in hours)	(±SD)	(in days^a^)
VSV-G	34.7	±0.0	0.7
Measles	14.1	±0.0	1.7
GALV	8.3	±1.0	2.9
Rabies	15.4	±1.0	1.6
RD114	21.2	±10.5	1.1
4070A	36.6	±2.7	0.7
10A1	13.8	±0.2	1.7
MuLV	18.6	±5.4	1.3

^a^The reduction factor based on the half-life of the envelope-pseudotyped lentiviral vectors is represented in days to be readily applicable in the formula for the Reduction Ratio of free infectious-lentiviral vector particles. For example: 34.7 h equals 1.4 days. Reduction factor per day: 1/1.4 = 0.7.

The half-life of the differently pseudotyped lentiviral vector particles ranged from around 8 to around 40 h and appears to depend, at least in part, on the envelope protein as the differences between the specific vectors were considerable. In our experiments, the half-life of VSV-G-pseudotyped lentiviral vector particles is around 35 h.

To exclude that the phenomenon of pseudotransduction [28] affect our transduction readings, we measured the GFP expression 48 h after transduction of the cells in the absence or presence of 20 and 50 µg/ml azidothymidine (AZT), a potent inhibitor of the HIV reverse transcriptase. AZT was found to block reporter gene expression, indicating that pseudotransduction does not affect the reported half-life values (data not shown).

### Cellular uptake of envelope-pseudotyped lentiviral vectors

The current applied estimation of the free lentiviral vector-particle concentration does not encompass vector loss due to infection of the target cells. First, we studied the effects of the infection protocol on the vector transduction and subsequently assessed whether the vector concentration that is retained in the culture medium after infection is decreased upon incubation with 293T or B77 cells. Typical transduction experiments using lentiviral vectors can be performed as stationary infection, where the cells with the vector-containing cell culture medium is incubated in a static manner at 37 °C/5% CO_2_, or alternatively using a ‘spin infection’ procedure. In the latter technique, the culture plate is centrifuged to spin the particles down onto the adherent cells. We performed a stationary and a spin infection with three of the envelope-pseudotyped lentiviral vectors under identical circumstances and compared both conditions by calculating the ratio between the percentage of GFP-positive cells in the spin infection by the percentage in the stationary infection (Tables 4–6).

**Table 4:** Increased cell transduction by lentiviral vectors upon spin infection.

Envelope protein	Spin infection^a1^		Stationary infection^a2^		Ratio spin/stationary infection^b^	
	% of GFP-positive cells, ±SD					
VSV-G	51.5	±2.8	20	±0.8	2.6	±0.2
Rabies	25.4	±0.6	17.7	±1.5	1.4	±0.1
4070A	58.8	±1.8	10.1	±0.3	5.8	±0.2

^a^The percentage of GFP-positive cells at 72 h post transduction with an envelope-pseudotyped lentiviral vector using either a spin (^a1^) or a stationary infection procedure (^a2^), ±SD represents technical triplicates.

^b^The ratio (±SD) of the percentage of GFP-positive cells between a spin and a stationary infection procedure upon transduction with envelope-pseudotyped lentiviruses.

**Table 5:** Reduction of free lentiviral vector concentration in inoculum by stationary or spin infection.

Envelope protein	1st infection—no cells^a^		1st infection—Stationary^b1^		1st infection—Spin^b2^		Ratio no cells/stationary infection^c^		Ratio no cells/spin infection^d^	
	% of GFP-positive cells in 2nd infection, ±SD						±SD		±SD	
VSV-G	17.8	±0.6	22.6	±0.6	6	±0.6	0.8	±0.0	3.0	±0.3
Rabies	21.1	±1.2	21.6	±1.8	19.2	±2.0	1.0	±0.1	1.1	±0.1
4070A	41.3	±5.5	31.9	±3.6	28.3	±3.1	1.3	±0.2	1.5	±0.3

^a^The percentage of GFP-positive cells at 48 h post transduction using a spin infection procedure, where the inoculum containing the envelope-pseudotyped lentiviral vector was first incubated overnight in the absence of cells (mock condition), ±SD represents technical triplicates.

^b^The percentage of GFP-positive cells at 48 h post transduction using a spin infection procedure, where the inoculum containing the envelope-pseudotyped lentiviral vector was first subjected to a stationary (^b1^) or spin (^b2^) infection procedure with cells, ±SD represents technical triplicates.

^c^The ratio (±SD) of the percentage of GFP-positive cells between ^a^ (first infection: no cells) and ^b1^ (first infection: stationary infection procedure).

^d^The ratio (±SD) of the percentage of GFP-positive cells between ^a^ (first infection: no cells) and ^b2^ (first infection: spin infection procedure).

**Table 6:** Reduction Ratio based on lentiviral vector particles in culture medium upon spin infection.

Envelope protein	Reduction percentage of GFP+ cells^a^		Reduction ratio^b^
	Spin infection versus no cells in 2nd infection, ±SD (%)		
VSV-G	88	±3	8.1
Measles	78	±11	4.5
RD114	58	±8	2.4
4070A	47	±7	1.9
10A1	87	±3	7.8
MULV	29	±3	1.4

^a^The average reduction (%) in the percentage of GFP-positive cells in a spin infection procedure compared with a mock infection procedure, ±SD represents technical triplicates (The inoculum containing the envelope-pseudotyped lentiviral vector was first subjected to a spin or mock infection and this culture medium was subsequently used in a spin infection).

^b^The reduction factor based on the reduction of infectious-lentiviral vector particles in the inoculum after a spin infection procedure (compared with a mock infection). The ratio is calculated by: the percentage of GFP-positive cells in the mock infection divided by the percentage of GFP-positive cells in the spin infection procedure.

From the data it became apparent that a spin infection with these three envelope-pseudotyped lentiviral vectors led to an increased percentage of GFP-positive cells compared to a stationary infection. The extend of the increase differed per pseudotyped lentiviral vector.

Importantly, these results also showed that the measured amount of vector particles in the initial inoculum of a transduction experiment was strongly affected by the experimental procedure used to determine the titer of the vector batch. Stationary infection procedures and, to a lesser extent, spin infection procedures tend to underestimated the amount of vector particles in the inoculum (see also Tables 4 and 5).

Another relevant parameter for the formula for the Reduction Ratio of free lentiviral vector particles is the potential decrease in free-vector concentration in the cell culture medium upon virus-uptake by cells. To address this, we transferred the culture medium of the infection described above to fresh cells and performed a second spin infection on each sample to maximize the amount of vector particles entering the cells. Next, we assayed the percentage of GFP-positive cells and calculated the ratio of GFP-positive cells between the vector-containing culture medium in the absence of cells from the first ‘infection’ (to correct for the half-life of the pseudotyped lentiviral particle) and the vector-containing culture medium from the first stationary infection. Also, we calculated the ratio of GFP-positive cells between the vector-containing culture medium in the absence of cells from the first ‘infection’ and the vector-containing culture medium from the first spin infection (Table 5).

The secondary infection demonstrated that the concentration of vector particles in the medium in a stationary infection did not significantly decrease by the initial infection. This is evident by the ratio of GFP-positive cells in medium that was incubated either with or without cells. The ratio between these conditions is around 1 for all assessed envelope-pseudotyped lentiviral vectors. For spin infections it appeared that there was, for some envelope-pseudotyped vectors, a decrease in vector concentration in the medium after a first infection round on cells. For VSV-G-pseudotyped lentiviral vectors this effect was most pronounced.

Next, we repeated the assay with seven of the envelope-pseudotyped lentiviral vectors for only the spin infection condition (Table 6). These data demonstrated a decrease in free-vector concentration in the medium upon a spin infection for all assayed envelope-pseudotyped lentiviral vectors, except for GALV (data not shown). The concentration of free GALV-enveloped-pseudotyped vector particles did not appear to be reduced after a spin infection. We did not investigate the cause of this deviant result for the GALV-envelope-pseudotyped vectors. Although all other envelope-pseudotyped vectors did show a reduced concentration in the culture medium after a spin infection, the differences between the different vectors were considerable and also the reduction factors between two experiments with the same envelope-pseudotyped vectors varied substantially (e.g., VSV-G-pseudotyped vectors showed a Reduction Ratio of respectively 3.0 and 8.1 in subsequent experiments). These data suggest that the inherent technical variations in the experimental procedures can affect the results. This hampers the establishment of a generalizable estimate for the reduction of the vector concentration in the culture media. However, this factor can be included in the Reduction Ratio formula if and when robust experimental data are provided to quantify the effect for a particular experimental setting.

## Discussion

Here, we determined a number of parameters that could be used for estimating the number of residual envelope-pseudotyped infectious-lentiviral vector particles. There are several factors that contribute to the decrease of the amount of viral vectors in the culture medium that are retained from the inoculum. Some of the factors are dependent on biological characteristics of the vector particles, while others are independent of the particles.

A vector-independent parameter is the efficiency with which vector particles are washed away with the culture medium of adherent cells. Upon removal of tissue culture medium with a plastic pipette or a platinum aspiration needle, a limited amount of medium remains and adheres to the culture dish. We assessed the total amount of liquid remaining in the dish by determining the dry weight of the dish, and after addition and the subsequent removal of the culture medium, the wet weight of the dish. Assuming random distribution of the viral vector particles in the liquid, this should give an estimate of the fraction of the vector that is retained in the dish after removal of the medium. We found that in two frequently used dish sizes (i.e., Ø35 mm, with a surface area equal to one well of a 6-well plate, and Ø10 cm diameter dishes) the remaining fraction was respectively 2% and 5%. The values were identical for the plastic pipette and the aspiration needle. This implies that removal of the medium reduces the amount of inoculum lentiviral vector particles by 20-fold on a Ø10 cm dish, and 50-fold on a Ø35 mm dish. Every subsequent washing step can be expected to have a similar reduction in remaining lentiviral vector-particle amounts. The 20-fold value is in agreement with the standard parameter proffered for the COGEM formula [20]. It would be prudent to allow researchers to determine the efficiency of washing in their culture system.

**Fig. 4:**
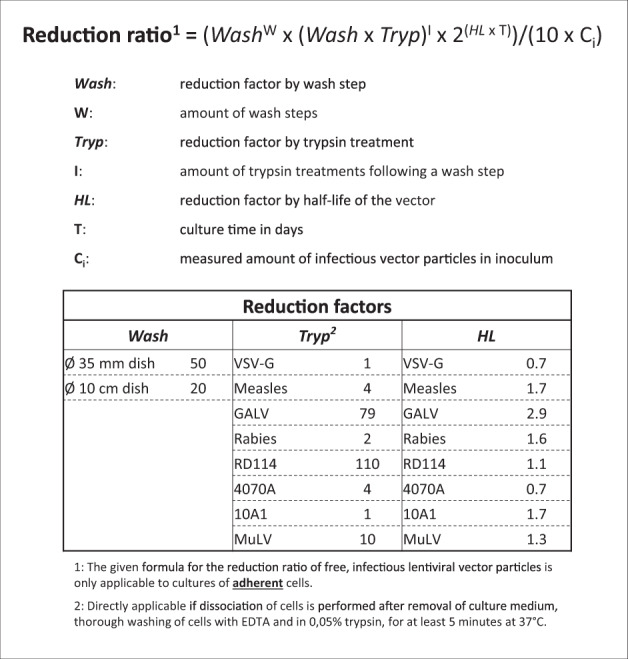
The new formula for calculation the Reduction Ratio of free infectious-lentiviral vector particles. In the formula the parameter ‘*Wash*’ represents the reduction factor upon a wash step of the transduced cells and ‘*W*’ signifies the number of times the cell culture was washed. The parameter ‘*Tryp*’ indicates the vector-dependent reduction factor for treatment of the cells with trypsin, where ‘*I*’ is the amount of trypsin treatments after a wash step. ‘HL’ is the reduction factor based on the half-life of the envelope-pseudotyped vector particles at 37 °C in culture medium and ‘*T*’ the total culture time in days since the transduction of the cells. The parameter ‘*C*_*i*_’ represents the measured amount of infectious vector particles in the inoculum. The formula calculates the Reduction Ratio, the fold-decrease in the concentration of free infectious vector particles relative to the amount of infectious viral vectors added to the cell culture.

A vector-dependent factor is the vector’s sensitivity to trypsin. Trypsin treatment is known to inactivate lentiviruses and VSV-G-envelope-pseudotyped lentiviral vectors [20, 29]. We determined the inactivation efficiency of seven envelope-pseudotyped lentiviral vectors. In the experiments, we used an amount of trypsin typically used to detach our cells from the culture dish (i.e., 0.05% trypsin-EDTA in PBS for 5 min at 37 °C). The efficiency differed substantially between the various envelope-pseudotyped lentiviral vectors and ranged from 1 to 100% residual vector infectivity after trypsin treatment. The residual activity value for VSV-G-envelope-pseudotyped particles (100% in 5 min) shows that trypsin inactivation for these vectors is less efficient than initially assumed on the basis of the trypsin inactivation data derived from wild-type HIV-1. It is therefore advisable to use the inactivation frequency from our study when applying the formula. This could be calculated as (Wash × Tryp)^*I*. In this formula, Wash represents the fold reduction of vector amount as result of washing, Tryp represents the fold reduction by standardized trypsin treatment as per this study, and *I* represents the number of trypsin treatments during the culture period for which the Reduction Ratio formula is applied (see also Fig. 4).

It has been described that envelope-pseudotyped lentiviral vector particles cannot only be inactivated by trypsin, but also by exposure to human complement [30, 31]. These studies demonstrate that human serum very efficiently inactivates VSV-G-pseudotyped lentiviral vectors but that, in contrast, lentiviral vectors pseudotyped with the amphotropic 4070A-envelope largely resist inactivation by human sera. This demonstrates that the efficiency of inactivation is dependent on the envelope protein. The same authors reported varying inactivation efficiencies with different serum batches. For VSV-G-pseudotyped lentiviral vectors the efficiencies of inactivation varied from 4 to 1000-fold for a 1 h incubation at 37 °C [31]. The donor-to-donor variability in the efficiency of complement-mediated vector inactivation hampers the use of this approach to inactivate free viral vector particles. It was also demonstrated that VSV-G-pseudotyped vectors could be inactivated by a heat-stable factor in the human serum. This led these authors to suggest that VSV-G-specific antibodies may be present in some batches of serum and these may contribute to the donor-to-donor variation in the serum inactivation efficiency.

The complement sensitivity is dependent on the cell line used to produce the vector particles. Takeuchi and collaborators demonstrated that Gal(alpha 1–3)Gal terminal carbohydrates are expressed by most mammals, but that these are absent in humans as humans lack a functional (alpha 1–3) galactosyltransferase gene [32]. Anti-Gal(alpha 1–3)Gal antibodies present in human serum can inactivate retroviruses produced from animal cells that express (alpha 1–3) galactosyltransferase. VSV itself had reduced stability in human sera when were grown on cells that are (alpha 1–3) galactosyltransferase positive [32, 33]. The sensitivity of vector particles produced in (alpha 1–3) galactosyltransferase positive cells to human serum may be dependent on natural IgM antibodies recognizing Gal(alpha 1–3)Gal in the human sera [34]. The inherent variations in the capacity to inactivate VSV-G-pseudotyped lentiviral vectors of human sera thwarts the definition of a robust rule-of-the-thumb estimate of the effectiveness of complement-mediated inactivation of lentiviral vectors. Also, the fact that the (alpha 1–3) galactosyltransferase status of the producer cells dictates the complement sensitivity is a complicating factor. Therefore it will be very difficult to reliably predict the impact of complement treatment on residual vector-particle concentrations as intended by the COGEM formula.

Lentiviral-vector particles have a finite half-life at 37 °C. Although it is unknown what step in the lentiviral vector transduction pathway is inhibited when the particles lose their capacity to transduce cells, it is tempting to speculate that this is governed primarily by the envelope protein. This is based on the observation that particles that have their envelope-pseudotyped with different heterologous envelope proteins vary in their half-lives. The half-lives of the various vectors are summarized in Table 3. Remarkable is the relative stability of the VSV-G-envelope-pseudotyped particles. A half-life of 34.7 h is considerably higher than the value of 10.4 h reported earlier [35]. We can only speculate on the reasons for the discrepancy, and we suppose it may be caused by differences in the buffer conditions used. Whereas we determined the half-life in standard bicarbonate-buffered tissue culture medium (i.e., DMEM supplemented with 8% FCS), these authors studied the stability in 100 mM TRIS-HCl buffer at pH = 7.0. Moreover, the latter authors in the same report also describe a half-life value of 321.2 min (5.35 h) using apparently very similar conditions [35].

We also noted that inclusion of polybrene, a polycation often included in the lentiviral vector transduction medium to enhance the infection efficiency, affected the half-life of VSV-G-envelope-pseudotyped lentiviral vector particles in culture medium. In the absence of polybrene in the culture medium a half-life value of 49.5 h was found, while in the presence of 8 µg/ml polybrene a value of 21.0 h was found. Note that these half-life values differ from the values we reported above. This is due to a difference in the methodology used (i.e., storing the samples at −20 °C for variable times which was done in the experiments assessing the effect of polybrene). The mechanism underlying the effect of polybrene on vector half-life is unknown, but it is tempting to speculate that the particles may have a higher tendency to aggregate as a consequence of the electrostatic neutralization of the viral membranes. This would reduce the apparent infectious-lentiviral vector-particle titer.

Usually polybrene is present for maximally one day following the addition of the viral vector preparation, after which the medium is replaced by medium without polybrene. We decided to use medium without polybrene for determining the stability of the viral vectors. On the one hand because the effect of polybrene on the half-life of VSV-G-pseudotyped vectors is not apparent if the vector particles are directly incubated with cells (i.e., without prior incubation of vector particles in culture medium in the absence of cells). This *t* = 0 condition is the regular method to start a transduction experiment. Polybrene is usually omitted from the medium during the subsequent culturing of transduced cells while free infectious-lentiviral vector particles could still be present. Moreover, determination of the half-life in absence of polybrene, which reduces the half-life of the vectors, represents the desired situation from a safety perspective, it could be seen as a ‘worst-case’ measurement.

For the viral vectors that were envelope-pseudotyped with other envelope proteins the stability was measured only in the absence of polybrene. For all of the envelopes the half-life varied between 8.3 and 36.6 h.

Currently, the physical titer of a lentiviral vector batch is routinely determined with a p24 ELISA assay, measuring vector associated and free p24 protein in a stock, which is converted to ng of vector per volume. The use of this method automatically implies an overestimation of the amount of physical particles in a stock. Moreover, batch to batch variations in the amount of free p24 protein in the solution complicates the reliability of the obtained physical titer. Furthermore, with the current assay methods it is extremely complex to convert the physical vector titer into an infectious vector amount. In the research field a general rule of thumb for VSV-G-pseudotyped lentiviral vectors is widely in use: the addition of 1 ng of p24 to 2500 cells is considered as an multiplicity of infection (MOI) of 1 (e.g., transduction of 100,000 cells with MOI 10 requires 400 ng of vector). This conversion factor was based on the values provided by literature data which demonstrated a range suggesting that 1 ng p24 is the equivalent of 1000–5000 infectious units [2]. We confirmed this and found a value of 1 ng p24 is the equivalent of 2500 infectious units by diluting a CMV-GFP virus-vector stock with a known amount of p24 on 293T cells. The conversion factor works reasonably well in experiments on most cell lines.

Besides the uncertainties in the current assays to titer a vector batch, also experimental variations in titration procedures may influence the resulting titer. Sometimes the infection efficiency of lentiviral vectors is enhanced by centrifugation (e.g., the spin infection procedure). For three vectors we determined the effect of a spin infection on the apparent titer of the vector stock. In 4070A-envelope-pseudotyped vectors the gain of infectious titer upon spin infection was 5.8-fold. In VSV-G-pseudotyped lentiviral vectors the ratio between the spin and non-spin infection was found to be 2.6-fold. For rabies envelope-pseudotyped particles this difference was less pronounced with a ratio of 1.4. While the reasons for these differences are unknown, the data imply that the amount of input vector could be underestimated considerably if based on a stationary vector titration experiment. This effect may be caused by the physical size of vector particles which severely limit Brownian motion of the vector particles [36]. The predominant manner in which the vector particles can move in the dish is by fluid convection, which is the movement of the fluid itself [36, 37]. The consequence of this is that the apparent titer of a vector suspension is strongly affected by the volume in which the experiment is performed. Doubling the volume but not the amount of virus can reduce the number of cells infected and thereby causing an underestimation of the apparent titer of up to twofold. The consequence is that the apparent titer of the lentiviral vector stocks may be a considerable underestimation of the actual amount of infectious-lentiviral particles. This is of importance as the titer is used in COGEM’s formula to calculate the Reduction Ratio. Given the 2.6 and 5.8-fold increase in apparent infectious viral particle titer, it seems reasonable to correct for this effect in the COGEM formula. This could be done by multiplying the measured amount of initial vector particles in the culture medium (*C*_*i*_) by 10. By this multiplication, the uncertainty in determining the exact amount of infectious-lentiviral vector particles in a stock solution can be largely intercepted.

Another factor that has been proffered as being neglected in the COGEM formula is the amount of vector that is taken up by the cells, and should (could) be subtracted from the amount of free-vector particles. We determined the residual amount of vector particles after infection using the spin infection procedure. Here we noted for most envelope-pseudotyped vectors a depletion as result of cellular uptake of infectious-lentiviral particles after a spin infection procedure (ranging from 0% (GALV) to 88% depletion), but the inter-experimental differences were substantial. Given the inherent technical variations in the procedure and the transduction efficiency of cells it seems unfeasible and unwarranted to provide a robust and generalizable parameter for estimating the reduction of viral vector titer as result of vector loss due to transduction of the target cells. Hence this factor cannot be included in the Reduction Ratio formula, unless the experimental data are provided which should be obtained with lentiviral vectors, cell systems, and infection procedure identical to those to be used in the studies for which permission is sought.

In this report, we provide new data for the stability of envelope-pseudotyped lentiviral vectors. These data can be used to estimate the number of residual infectious-lentiviral vector particles after infection of cells in culture. The approach for calculation of the maximal amount of free-vector particles via the COGEM formula is broadly applicable. However, in the COGEM-formula approach a notable exception is made for macrophages and dendritic cells. These cells can take up lentiviral vectors and release them several days later in an infectious form [20]. Washing and trypsinization of such cell cultures is therefore ineffective for removing internalized infectious vector particles. Hence the trypsinization and the washing steps cannot be taken into account. This mechanism was described for the HIV virus itself, and to the best of our knowledge not (yet) for envelope-pseudotyped lentiviral vectors [38]. Reversely, we are not aware of data disputing this internalization phenomenon for lentiviral vectors, and therefore, this exception should be kept in place. Handling lentivirus-transduced macrophages and dendritic cells therefore requires a case-by-case risk analysis.

The amount of free infectious vector particles remaining from the inoculum is of importance as these particles, together with replication-competent lentiviral vectors potentially generated during production with early generation production systems, and the resulting vector-modified cells constitute the three components that should be considered in the risk analysis that must precede any use of lentiviral vectors.

While the presence of replication-competent lentiviruses in the vector stocks can be excluded with the new production systems available to date, and the vector-modified cells have readily predictable phenotypes, the amount of the residual infectious replication-defective viral vector particles that remains from the inoculum needs to be quantified. However, it may be difficult to experimentally determine this number in the cell product. As a guide COGEM offered a formula to estimate the Reduction Ratio and the maximal number of residual infectious-lentiviral particles in the cell product [20].

In this study, we evaluated the current COGEM formula and generated new data for VSV-G-envelope-pseudotyped lentiviral vectors and particles with seven other heterologous envelope proteins to be used as variables in the formula. Moreover, we propose to adapt the formula to correct for the underestimation of infectious titer of the inoculum by multiplying the amount of initial vector particles in the culture medium (*C*_*i*_) by 10. Together, these alterations lead to a more accurate formula for calculating the Reduction Ratio (Fig. 4).

If the Reduction Ratio exceeds the desired value, than the presence of infectious-lentiviral vector particles remaining from the inoculum can be safely excluded. If the desired Reduction Ratio is not achieved, then a formal risk analysis dedicated to risks associated with the presence of such particles is required, or alternatively, precautions should be taken under the assumption that lentiviral particles may be present. While this may seem rather rigid, it fair to say that it is difficult to conceive realistic scenarios on the inadvertent exposure to small amounts of replication-defective lentivirus-vector particles that would lead to notable risks to the experimenters. This is even more so if strictly the environmental risks are considered, i.e., the risks beyond experimenters handling lentiviral-vector-modified cell products in a contained-use setting, or beyond the patients receiving such cells as medicinal products in a deliberate release setting. In a deliberate release setting there could be free infectious-lentiviral vector particles in the cell product that is administered to a patient. The likelihood that these free particles are shed and passed to third persons seems minute [39]. Also, the hazards associated with infectious vector particles being passed to third persons appear minimal, as it can be assumed that it would concern very small quantities. Additionally, we are not aware of any literature data demonstrating a direct harmful effect of unintended exposure to SIN lentiviral vector lacking a harmful insert. However, it should be noted that the availability of literature data on this topic is limited. This lack of data about possible scenarios demands caution and careful evaluation of individual applications using lentiviral vectors in a deliberate release setting. Taken together, the use of lentiviral vectors can be considered safe, even if products are handled that contain replication-defective infectious-lentiviral vector particles. That being said, gene therapists should, at all times, aim for a minimal amount of free infectious-lentiviral vectors particles in their products. As lentiviral vectors increasingly become the tool of choice for gene transfer, this offers excellent opportunities for collecting more data on using these new therapeutic entities in daily practice.
